# Effects of Cervical Spine Exercise Protocol on Neck Pain, Pericervical Muscle Endurance, and Range of Motion in Medical Students: A Prospective Study

**DOI:** 10.7759/cureus.27160

**Published:** 2022-07-22

**Authors:** Bryan G Anderson, Brett Benzinger, Jason Chickness, Chris Hietanen, Kylan Hill, Jean-Marc P Lucas, Joshua Tuck, Michael Ghassibi

**Affiliations:** 1 Department of Spine Surgery, Swedish Neuroscience Institute, Seattle, USA; 2 Department of Orthopedic Surgery, Lake Erie College of Osteopathic Medicine Health, Erie, USA; 3 Department of Osteopathic Medicine, Lake Erie College of Osteopathic Medicine, Erie, USA; 4 Department of Radiology, University at Buffalo, Buffalo, USA; 5 Department of Obstetrics and Gynaecology, Summa Health, Akron, USA; 6 Department of Osteopathic Medicine, Lake Erie College of Osteopathic Medicine, Bradenton, USA; 7 Department of Orthopedics, Lake Erie College of Osteopathic Medicine Health, Erie, USA; 8 Department of Orthopedics, Orthopedic Associates of Meadville, Meadville, USA

**Keywords:** nonoperative, neck strengthening, neck stretching, cervical spine exercise, neck pain

## Abstract

Introduction

Neck pain is a common and debilitating ailment that places a significant burden on the healthcare system. No practical protocols have been published utilizing a portable, commercially available, and affordable device that significantly reduces acute and chronic neck pain.

Methods

Forty-six young adults with or without mild-to-moderate neck pain completed a six-week neck stretching and strengthening protocol with a portable cervical stretching and strengthening device. The primary outcome was changes to pericervical muscle endurance. Secondary outcomes were changes to cervical range of motion (ROM), neck length, circumference, and subjective pain, flexibility, and strength. Measurements were obtained on study days 0, 21, and 42.

Results

A significant increase in pericervical muscle endurance was demonstrated across all planes of cervical motion, ranging from 84% to 105%. Cervical ROM improved across all planes of motion but was only significant in right-side bending (5.3°), left rotation (6.2°), and right rotation (7.8°). Subjective pain evaluated via the Numeric Rating Scale (NRS) saw statistically significant improvement as well (1.33 to 0.51). Subjective assessment of participant cervical pain, strength, and flexibility improved 61.3%, 95.7%, and 97.8%, respectively.

Conclusions

A six-week pericervical muscle stretching and strengthening program increased pericervical endurance and ROM in young adults. Decreased cervical pain was seen using the NRS and modified pain scale across most participants.

## Introduction

Neck pain is a condition that affects many at some point in their lives, with an incidence of 21%, and a 12-month prevalence between 30% and 50% [1,2]. Chronic neck pain patients utilize healthcare services twice as frequently as the average population [3]. Risk factors for developing neck pain in the adult population include genetics, exposure to tobacco, manual labor occupations, over-time work, high mental workload, poor psychological health, and unsatisfactory leisure time in the adult population [2,4-6].

It has been shown that conservative treatment involving active pericervical stretching and strengthening has short- and long-term benefits in reducing neck pain and improving function [7-9]. Some of these pericervical muscles include levator scapulae, rhomboid, sternocleidomastoid, trapezius, splenius, semispinalis, and multifidus. Pharmacologic and manual provider-directed mitigation strategies (i.e. osteopathic, chiropractic, and physical therapy techniques) have been extensively described [10-14]. Although these strategies are often sought out by patients experiencing neck pain, similar readily available self-mitigation methods to resolve mild-to-moderate neck pain are often not practical and lack high-level evidence [15-19]. This study aims to determine whether a novel commercially available device that was created for individuals to use ad hoc in any environment (e.g. their home, place of work, etc.) is effective at increasing pericervical muscle endurance and cervical range of motion (ROM), thus decreasing mild-to-moderate subjective neck pain. The present study focuses on treating neck pain in medical students because this population contains prevalent neck pain due to relatively benign pathology.

## Materials and methods

Participants

Two hundred one medical students ≥18 years (range 21-31 years) old with varying neck pain based on the Numeric Rating Scale (NRS) were recruited for study inclusion in August 2019. Of the 201 volunteer participants, 50 subjects (30 male, 20 female) were randomly selected for study inclusion through random number generation with the utilization of Microsoft Excel in a simple randomization. Subjects were screened by an orthopedic surgery resident (BA) at the time of initial data collection. Inclusion criteria included mild-to-moderate cervical pain based on the NRS and acute, subacute, or chronic onset of pain. Exclusion criteria included age <18 years at the start of the study, lifetime history of cervical pathology diagnosis, surgery, trauma within the last six months, severe cervical pain, moderate-to-severe pain with ROM exercises, radicular pathology, disc pathology, and facet pathology. Radiographs used for exclusion criteria were first read by a radiologist with a musculoskeletal subspecialty and then confirmed by the principal investigator (BA). The study was approved by the appropriate institutional review board: protocol 26-064. All subjects gave written informed consent prior to participation.

Study design

This was a single-arm prospective cohort in which subjects without known cervical pathology were evaluated for change in pericervical muscle endurance, cervical ROM, neck circumference, and subjective pain over six weeks while using a 10-minute-per-day cervical stretching and strengthening protocol designed to be used over 42 days with the NeckX® device (NeckX®, LLC, Aspen, CO). NeckX® is not a medical device, but rather a strength and conditioning device and therefore does not require Food and Drug Administration (FDA) approval for sale or use. Pericervical muscle endurance for the sake of this study is defined as the pericervical musculature's ability to resist fatigue following repetitive isotonic contraction. Data regarding subject pericervical muscle endurance, ROM, neck circumference, and subjective pain were recorded immediately prior to study commencement (i.e. study day 0) and on study days 21 and 42. Pre-study and post-study questionnaires in addition to NRS for pain reporting were completed on study days 0, 21, and 42. Subject data were desensitized and stored on an encrypted database accessible only by the research team. Study subjects and research team members were blinded to previous measurement data during inter-/post-study data collection and analysis.

Clinical evaluation

**Figure 1:**
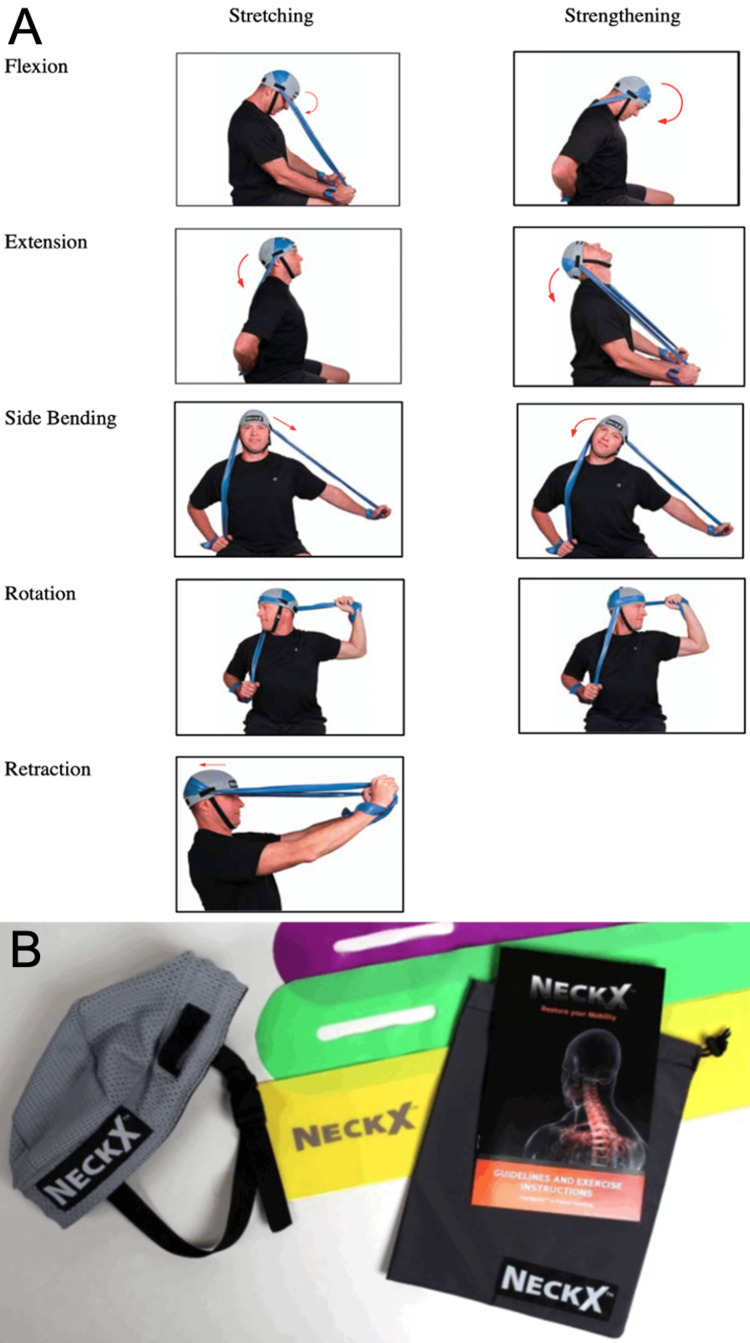
Neck stretching and strengthening protocol with NeckX® device. A) Demonstrates stretching and strengthening movements. B) Demonstrates NeckX® device including elastic bands. Used with permission from NeckX®.

Pericervical muscle endurance was the primary outcome criterion and was assessed using the NeckX® device on study days 0, 21, and 42. For each strengthening exercise, cervical flexion, extension, side bending (bilaterally), and rotation (bilaterally), subjects completed as many full repetitions as they could in 60 seconds. Subjects were allowed to rest for 60 seconds between each exercise. Device utilization and proper form were demonstrated for participants prior to utilization of the protocol and were adhered to for each strength assessment. Subjects demonstrated proper exercise form to the research team before initiating the at-home stretching and strengthening workout protocol (Figure 1A). The NeckX® device is shown in Figure 1B.

Cervical ROM was a secondary outcome criterion and was assessed using a head-mounted Halo digital goniometer (Halo Medical Devices, Sydney, Australia) on study days 0, 21, and 42. Subjects stood in a neutral spine position and then three repetitions of cervical flexion, extension, side bending (bilaterally), and rotation (bilaterally) measurements were obtained and averaged. For each motion, the subjects were asked to move their head as far as they could in that plane of motion three times without involving the thoracic vertebrae.

Neck circumference and length were secondary outcome criteria and were recorded on study days 0 and 42. For circumference measurements, subjects stood in a neutral spine position and circumference was measured at two points: mid-neck and base of the neck. Mid-neck circumference was measured by placing the superior border of the tape measure just below the laryngeal prominence and perpendicular to the long axis of the neck. Base-of-neck circumference was measured by placing the superior border of the tape measure just below the spinous process of C7 and perpendicular to the long axis of the neck.

Subjective assessments of cervical pain, flexibility, and strength were secondary outcome criteria and were obtained on study days 0, 21, and 42. The pain was assessed using both the NRS for pain and a modified pain scale that reported neck pain as “worse,” “the same,” or “improved.” Cervical strength and flexibility were assessed using a 10-point analog scale: 0 = no change; 10 = extremely improved.

Study protocol

The 46 subjects who completed the study were present for the three data collection events (Table 1). The workout protocol involved a 10-minute workout, five days per week over six weeks that was completed at the subject’s location of choice (e.g. home, gym, or school). The workout involved 3 minutes of static cervical stretching and 7 minutes of isotonic cervical strengthening activities. The workout was a progressive protocol involving increased repetitions and stronger therapy bands as the subjects continued through the six-week protocol.

**Table 1:** Workout protocol with stretching and strengthening device.

	Week 1	Week 2	Week 3	Weeks 4, 5, and 6
Therapy band color	Yellow	Green	Blue	Blue
Stretching (1 rep = 10 s of passive stretch)				
Flexion	2 reps	2 reps	2 reps	2 reps
Extension	2 reps	2 reps	2 reps	2 reps
Side bending	2 reps (right)	2 reps (right)	2 reps (right)	2 reps (right)
	2 reps (left)	2 reps (left)	2 reps (left)	2 reps (left)
Neck rotation	2 reps (right)	2 reps (right)	2 reps (right)	2 reps (right)
	2 reps (left)	2 reps (left)	2 reps (left)	2 reps (left)
Strengthening				
Flexion	10 reps x 2 sets	10 reps x 2 sets	10 reps x 2 sets	15 reps x 2 sets
Extension	10 reps x 2 sets	10 reps x 2 sets	10 reps x 2 sets	15 reps x 2 sets
Retraction	10 reps x 2 sets	10 reps x 2 sets	10 reps x 2 sets	15 reps x 2 sets
Side bending	10 reps x 2 sets (right)	10 reps x 2 sets (right)	10 reps x 2 sets (right)	15 reps x 2 sets (right)
	10 reps x 2 sets (left)	10 reps x 2 sets (left)	10 reps x 2 sets (left)	15 reps x 2 sets (left)
Rotation	10 reps x 2 sets (right)	10 reps x 2 sets (right)	10 reps x 2 sets (right)	15 reps x 2 sets (right)
	10 reps x 2 sets (left)	10 reps x 2 sets (left)	10 reps x 2 sets (left)	15 reps x 2 sets (left)

Statistical analysis

Clinical values of pericervical muscle endurance, cervical ROM, neck circumference and reported subjective cervical pain, strength, and flexibility were statistically analyzed. Statistical comparison was made between study days 0, 21, and 42 for each respective category using a two-way analysis of variance (ANOVA) with multiple comparisons, followed by a Tukey-Kramer significant difference test applying the Holm-Sidalk method. An alpha of 0.05 was utilized for all tests. Statistical analysis alpha value of 0.01 was set according to the Sidalk correction of Bonferroni inequality. Subjective cervical responses were analyzed using chi-squared analysis with an alpha of 0.05. Analysis was performed using GraphPad Software, LLC, version Prism 8.

## Results

Subject demographics

Forty-six subjects (28 male, 18 female) completed the study with one subject withdrawing prior to study day 21 due to increased cervical pain associated with the study (which resolved upon cessation of the workout protocol). Three subjects were removed from the study due to completing <75% of the protocol. Subject demographics are shown in Table 2.

**Table 2:** Subject demographics. BMI: body mass index; SD: standard deviation.

Subject Demographics	Study Group
Number of subjects	46
Age in years (range)	24 (21-31)
Sex (percent of total)	
Male	28 (61%)
Female	18 (39%)
BMI in kg/m^2 ^(±SD)	25.8 ± 8.18

Pericervical muscle endurance

**Figure 2:**
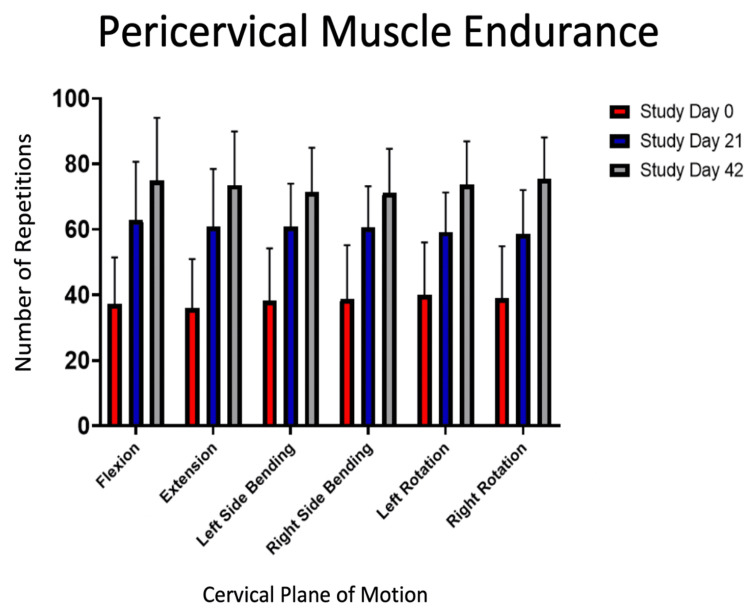
Summary of changes to pericervical muscle endurance. Significant increases were seen in all cervical planes of motion on study day 21 and study day 42 compared to study day 0 (p < 0.0001).

All subjects achieved a significant increase in their pericervical muscle endurance (p < 0.0001) across all cervical planes of motion (Table 3, Figure 2). Total improvement in pericervical muscle endurance ranged from 84% (right-side bending) to 105% (extension).

**Table 3:** Pericervical muscle endurance changes over time. Values given here represent means ± standard deviation.

Plane of Motion	Study Day 0 (repetitions)	Study Day 21 (repetitions)	Study Day 42 (repetitions)
Flexion	37.30 ± 14.18	62.87 ± 17.78	74.98 ± 19.11
Extension	35.95 ± 15.00	60.91 ± 17.52	73.52 ± 16.38
Left-side bending	38.14 ± 16.06	60.85 ± 13.12	71.40 ± 13.56
Right-side bending	38.61 ± 16.57	60.52 ± 12.63	71.14 ± 13.52
Left rotation	40.05 ± 15.99	59.22 ± 12.07	73.71 ± 13.19
Right rotation	39.05 ± 15.85	58.65 ± 13.37	75.43 ± 12.67

Cervical range of motion

**Figure 3:**
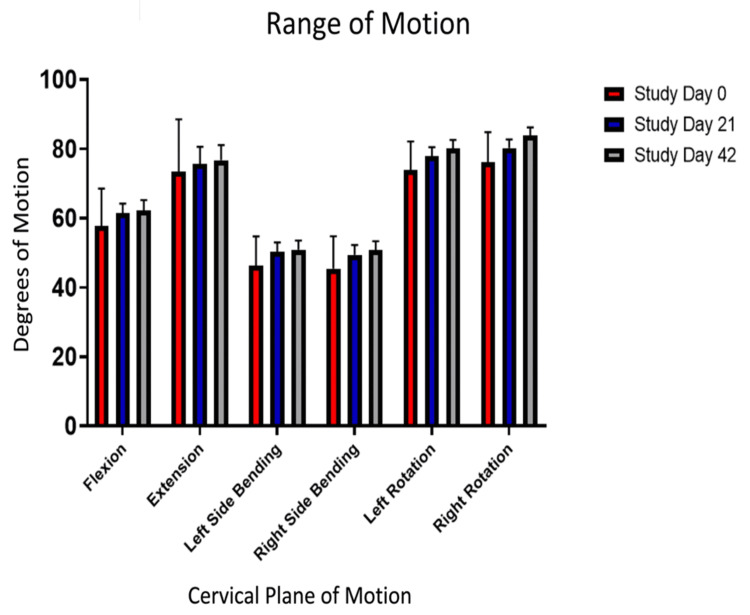
Summary of changes to pericervical range of motion. Significant increase in the degrees of active range of motion of the cervical spine was found between study day 0 and study day 42 for left rotation, right rotation, and right-side bending (p < 0.008).

All subjects saw a significant increase in ROM (p < 0.008) in right-side bending (5.3°), right rotation (7.8°), and left rotation (6.2°) (Figure 3). Improvements in left-side bending (4.7°) and flexion (4.6°) were not significant. Table 4 summarizes the ROM data.

**Table 4:** Cervical range of motion changes over time. Values given here represent means ± standard deviation.

Plane of Motion	Study Day 0 (degrees)	Study Day 21 (degrees)	Study Day 42 (degrees)
Flexion	57.64 ± 10.88	61.55 ± 8.90	62.20 ± 10.07
Extension	73.36 ± 15.17	75.66 ± 16.55	76.74 ± 14.65
Left-side bending	46.24 ± 8.47	50.18 ± 9.43	50.91 ± 8.89
Right-side bending	45.44 ± 9.32	49.40 ± 9.57	50.71 ± 8.84
Left rotation	73.83 ± 8.31	77.93 ± 8.58	80.03 ± 8.60
Right rotation	76.09 ± 8.74	80.25 ± 8.34	83.93 ± 7.61

Neck circumference

Neck circumference was measured on study days 0 and 42. There was no significant difference found in neck circumference at the base of the neck (p = 0.969) or the mid-neck (p = 0.515).

Subjective perception and satisfaction

Apart from one subject who withdrew due to increased pain prior to study day 21, no other subjects reported an increase in pain or a decrease in strength or flexibility associated with this study’s protocol.

**Figure 4:**
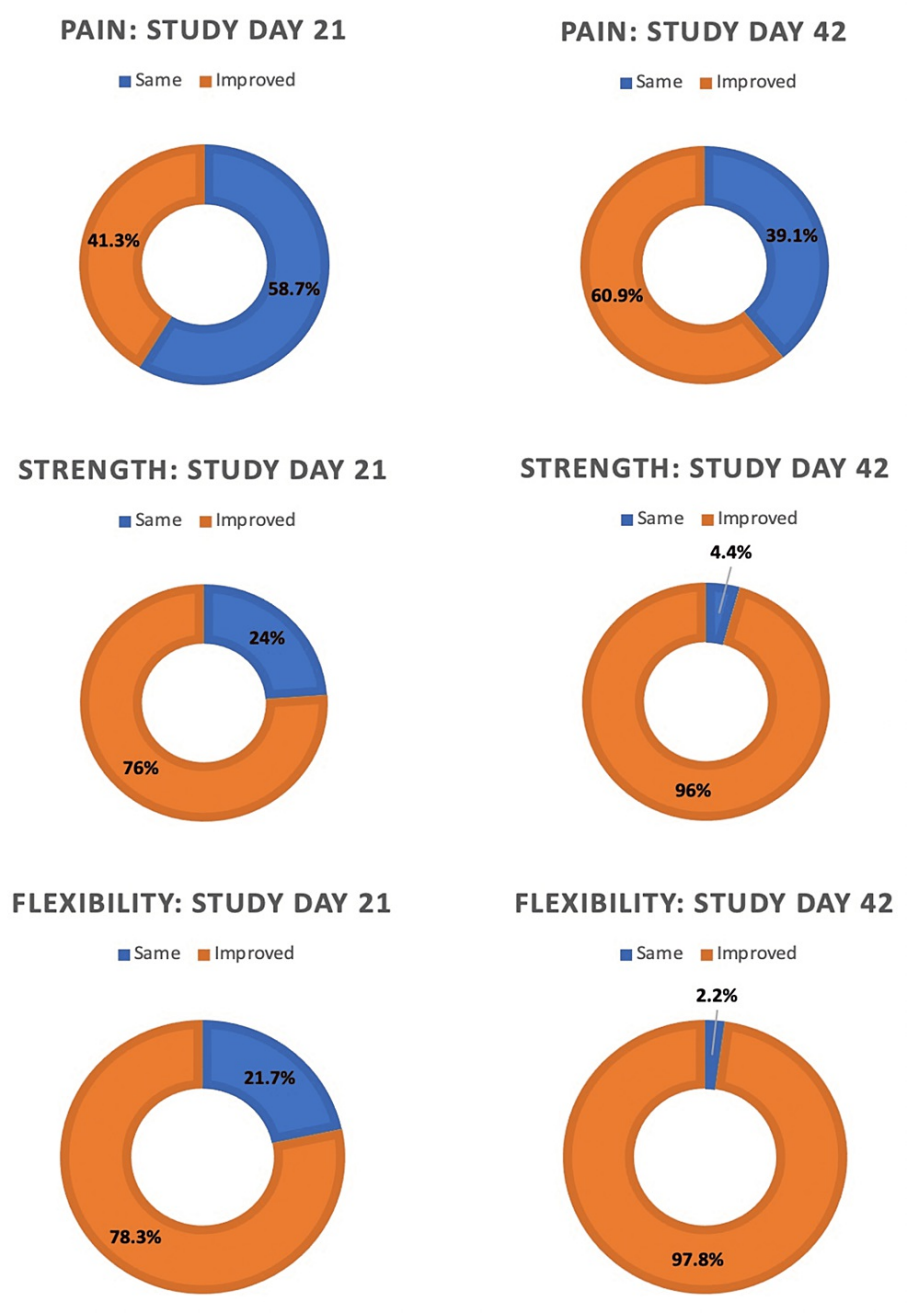
Subject perceived improvements of neck pain, strength, and flexibility. Significant improvements were noted for neck pain (p < 0.01), strength (p < 0.01), and flexibility (p < 0.01) between study days 0 and 21. Similar improvements were seen between study day 0 and 42 (p < 0.001, p < 0.001, and p < 0.001, respectively).

**Pain**: On study day 0, 58.6% of subjects reported experiencing mild-to-moderate neck pain. On study day 21, 41.3% of the subjects reported decreased neck pain compared to baseline (Figure 4; p < 0.01). On study day 42, 61.3% of subjects reported decreased neck pain compared to baseline (p < 0.001). The NRS pain scale showed significant improvement (p < 0.018) from study day 0 (mean = 1.33) to study day 42 (mean = 0.51).

**Strength**: On study days 21 and 42, 76.1% (p < 0.01) and 95.7% (p < 0.001) of subjects reported subjective increases in neck strength compared to baseline, respectively (Figure 4).

**Flexibility**: On study days 21 and 42, 79.3% (p < 0.01) and 97.8% (p < 0.001) of subjects reported subjective improvements in flexibility compared to baseline, respectively (Figure 4).

## Discussion

This study sought to validate a home-based pericervical muscle endurance and stretching protocol using a portable, commercially available, and economical device, which reduces neck pain through a daily 10-minute protocol lasting six weeks. Previous literature has demonstrated a positive correlation between improved pericervical muscle strength and decreased neck pain [7-9]. This study affirmed a positive association between pericervical muscle endurance and neck pain. These results validate this study’s protocol as an effective tool to reduce neck pain over a condensed therapeutic window.

Cervical ROM significantly improved in three planes. We theorize that ROM did not improve in all planes tested due to lack of ROM pathology in the subject population, as opposed to protocol inadequacy.

No significant change was noted in neck circumference over the six-week protocol, indicating that the amount of pericervical muscle endurance necessary to improve neck pain can be achieved without significantly increasing neck girth.

While the efficacy of pericervical muscle rehabilitative programs has not been extensively studied in the treatment of chronic neck pain, the literature has long supported treatment of lumbar pathology and associated chronic low back pain with stretching and strengthening of the lumbar and lower extremity musculature [20-23]. Lee et al. [20] demonstrated that subjects with chronic low back pain had significantly weaker trunk and knee extensor strength compared to age- and sex-matched controls. A 12-week physical rehabilitation program on trunk and knee extensor muscles in subjects with chronic low back pain has shown to significantly improve trunk and knee extension strength while decreasing pain and disability [21,22]. Nelson et al. [23] treated 46 patients indicated for lumbar or cervical surgery with a 10-week strengthening regimen; only three patients went on to receive the previously indicated surgery. With regard to the cervical spine, no succinct and convenient protocols have been published utilizing a portable, commercially available, and economical device that significantly reduces acute and chronic neck pain.

A study done by Ylinen et al. [8] demonstrated comparable results to this study with regard to neck pain, cervical ROM, and pericervical muscle endurance. Ylinen et al. found statistically significant improvement in neck pain, ROM, and endurance in middle-aged women who completed home-based neck stretching and isometric strengthening exercises with a Theraband (Hygiene Corp, Akron, OH) three times weekly for one year. In contrast, this study found improvements in these same parameters with only three to six weeks.

Highland et al. [24] reviewed the effectiveness of a machine-based (MedX Cervical Extension Machine; MedX Corp., Ocala, FL) cervical flexion and extension strengthening program on pericervical muscle strength, ROM, and perceived neck pain. Their patient populations included subjects with documented cervical strains, degenerative disc, and herniated disc diseases (n = 90). Subjects engaged in 12 gym-based workout sessions across eight weeks. The authors noted significant improvement in strength, ROM, and neck pain across the cohort and in all diagnosis groups. While effective, this study’s exercise machine-based protocol may be cumbersome for the general population.

Häkkinen et al. [25] studied the effects of manual therapy and stretching on neck pain and cervical strength and ROM in a randomized control trial of 125 women with chronic neck pain diagnosed as cervical strains. Subjects engaged in either active stretching and manual therapy (five times weekly for four weeks) or manual therapy alone (twice weekly for four weeks). The authors found statistically significant improvements for both groups in neck pain and cervical ROM. However, only mild improvements were seen in both groups with regard to pericervical muscle strength. These findings bring in to question the long-term neck pain reduction benefit of stretching and manual therapy protocols when pericervical muscle strengthening is excluded.

This study’s improvements in pericervical muscular endurance, ROM, and perceived neck pain measurements are promising because they were observed while utilizing a considerably more concise (three-to-six week) exercise protocol than previously mentioned studies. The short protocol (10 minutes per day, five days per week for six weeks) and portable design allowed participants to complete the exercise protocols at their own leisure throughout the day instead of having to go to a gym or fitness center to perform their daily routine, which likely led to increased compliance. It is also likely that by incrementally increasing the resistance of the exercises on study days 14 and 21 ROM improved more rapidly when compared to previously discussed protocols which took months to see improvements.

Limitations

The design of this study was limited by the population. Although standard random sampling techniques were utilized in the selection of the participants, this study was designed to evaluate young graduate students within a given geographical location. This resulted in a population that was between the ages of 21 and 31 and may not accurately depict normal healthy populations, let alone diseased populations. Future studies are required to validate this protocol and device in older and diseased populations.

Another study limitation was the lack of a control group. Although all subject data at study days 21 and 42 were compared to baseline data obtained on study day 0, the use of a control group or a separate study arm utilizing a different treatment intervention would provide further strength to the data that were obtained in this study.

The brevity of the study and short follow-up can also be criticized. This study did not follow up with subjects at longer intervals to assess continued voluntary compliance to the protocol, long-term pericervical muscle endurance, and ROM or the lasting improvement or worsening of the subject’s neck pain.

## Conclusions

Both active ROM and endurance of the pericervical muscles increased following our incremental six-week training protocol. Furthermore, a majority of the subjects reported a significant improvement in their day-to-day cervical neck pain after undergoing six weeks of pericervical muscle stretching and endurance training. Thus, it is the authors’ belief that this protocol may delay or negate surgical treatment and provide substantial benefit to subjects with significant pain and advanced cervical disease (e.g. history of chronic cervical muscle strains, cervical facet arthrosis and/or herniated disc disease with concomitant neck pain). Future studies should be performed with this subject group to validate the efficacy of this inexpensive, convenient device and novel protocol. This could be a powerful conservative tool for patients, therapists, primary care providers, and specialists. The authors postulate that this training protocol may be an effective prophylactic workout regimen for high-risk athletes. As such, future studies should also focus on high-impact sport athletes (e.g. soccer, football, hockey, lacrosse, etc.) and the ability of this device and protocol to prevent and treat sport-related pericervical injury, neck pain, ROM deficits, and possibly concussion.
